# On-filter derivatisation and alkaline trap sampling for UAV-based gas-phase halogen speciation in volcanic plumes

**DOI:** 10.1007/s00216-026-06403-7

**Published:** 2026-02-26

**Authors:** Bastien Geil, Nicole Bobrowski, Thorsten Hoffmann

**Affiliations:** 1 Department of Chemistry, Johannes Gutenberg-University, 55128 Mainz, Germany; 2 Institute of Environmental Physics, Ruprecht-Karls-University, 69120 Heidelberg, Germany; 3 Istituto Nazionale Di Geofisica E Vulcanologia, 95125 Osservatorio Etneo, Catania Italy

**Keywords:** Volcanic gases, Molecular halogens, Interhalogens, Bromine chloride, Alkaline trap, GC–MS

## Abstract

Volcanic halogen emissions play a critical role in atmospheric chemistry and serve as valuable indicators of volcanic activity. However, direct in situ quantification and speciation of reactive halogen species (RHS) remain analytically challenging, particularly under the constraints of UAV-based sampling. In this study, we present a suite of miniaturised and field-deployable methods for halogen speciation in volcanic plumes, optimised for unmanned aerial vehicle (UAV) platforms. Two types of coated syringe filters, based on cis-stilbene and 1,3,5-trimethoxybenzene (TMB), enable in situ derivatisation and selective detection of molecular halogens and interhalogens (e.g. Cl_2_, BrCl, Br_2_, I_2_). An alkaline trap system (“Bubbler”) was also developed for total halogen and sulphur quantification. Method performance was validated using a custom-designed chlorine permeation source in controlled laboratory and dilution chamber experiments. Detection limits were sub-ng for GC-HRMS (gas chromatography–high-resolution mass spectrometry)-based speciation and sub-µg for ion chromatography of total halogens. These results demonstrate the feasibility of quantitative, UAV-based halogen speciation in volcanic plumes, offering new possibilities to enhance our understanding in volcanic gas chemistry and atmospheric trace gas analysis.

**Graphical abstract:**

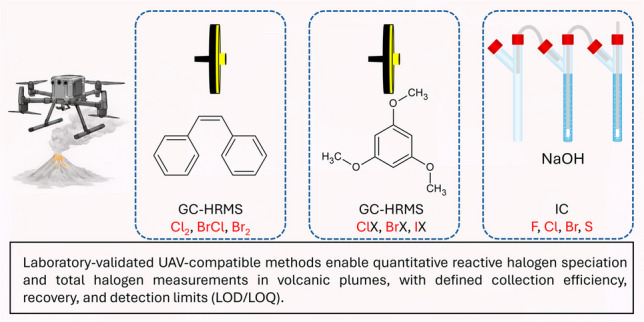

**Supplementary Information:**

The online version contains supplementary material available at 10.1007/s00216-026-06403-7.

## Introduction

Volcanic emissions represent a significant natural source of atmospheric trace gases, influencing both regional air quality and global climate. While major volcanic gas constituents such as H_2_O, CO_2_, and SO_2_ dominate in terms of abundance, reactive halogen-containing trace gases strongly influence atmospheric oxidation capacity, ozone chemistry, and aerosol formation, even at trace levels [1–3]. Moreover, the composition of volcanic plumes provides valuable information on magmatic processes and can serve as an early warning indicator of volcanic unrest. Thermodynamic modelling of volcanic gas emissions further supports the key role of halogens in trace element transport, indicating that many metals are likely present as chloride or mixed hydroxy-chloro species [4].

Hydrogen halides (HF, HCl, HBr, HI) are typically the most abundant halogen species released [5, 6]. These acidic gases, along with their oxidised counterparts, such as molecular halogens (Cl_2_, Br_2_, I_2_) and interhalogens (BrCl, ICl), contribute to complex multiphase chemistry in the troposphere. The detection of bromine monoxide (BrO) in volcanic plumes has highlighted the importance of in-plume oxidation pathways, driven by interactions with oxidants entrained from the atmosphere [3, 7–10]. Such processes can initiate autocatalytic halogen activation cycles, with implications for ozone depletion [11, 12]. However, reactive halogen species might also be used as volcanic activity proxies [13, 14] similar to traditional gas ratios like CO_2_/SO_2_ or SO_2_/HCl [15–18]. Although bromine chemistry has received most of the attention, iodine and chlorine also contribute to reactive halogen chemistry in volcanic plumes. For example, IO was detected via satellite in the plume of Mt. Kasatochi [19], and other species like OClO [20, 21], ClO [22] or Cl_2_ [23] have also been measured.

While remote sensing techniques—such as Differential Optical Absorption Spectroscopy (DOAS)—have been widely employed to quantify selected halogen species (e.g. BrO, IO) [24, 25], many key reactive intermediates remain undetectable with current optical methods although they have been predicted and incorporated into chemistry models [10, 21, 26, 27]. Moreover, remote sensing techniques, particularly optical spectroscopy, do not generally provide access to the full halogen budget. While HF is essentially chemically inert and thus a reliable measure of total fluorine, and HCl often serves as a reasonable proxy for total chlorine, this is not the case for bromine and iodine. For these elements, the primary emitted HBr and HI cannot be detected by field-deployable remote sensing instruments and rapid plume chemistry redistributes the emitted halogens among multiple reactive species. Ground-based direct sampling studies have long provided detailed insights into volcanic gas compositions and sampling strategies, as summarised in comprehensive reviews of fumarolic gas investigations [28]. Building up on these foundation, direct sampling approaches, such as alkaline traps and denuder systems [29–34], offer complementary capabilities by enabling quantitative collection of both reduced and oxidised halogen species.

Recent advances in UAV technology now allow aerial sampling in hazardous or logistically challenging environments. This study presents two complementary UAV-compatible sampling systems: (1) a miniaturised alkaline trap (“Bubbler”) for quantifying total halogen and sulphur content, and (2) in situ derivatisation filters coated with either cis-stilbene or TMB, enabling selective detection of specific RHS based on electrophilic addition or electrophilic aromatic substitution. The combination of field-deployable sampling and high-resolution analytical techniques (gas chromatography–high-resolution mass spectrometry (GC-HRMS) and ion chromatography (IC)) offers improved sensitivity and speciation capability for halogen analysis in volcanic plumes. This approach extends previous chromatographic developments, as comprehensively reviewed in [35], highlighting the use of GC and LC methods in volcanic gas surveillance. To characterise filter reactivity and validate method performance under controlled conditions, a novel chlorine permeation source was developed.

The purpose of this paper is to present the development and validation of drone-based analytical methods for the in situ speciation of gaseous halogens in volcanic plumes, enabling spatial and temporal characterisation of reactive halogen compounds under challenging field conditions. These tools extend the analytical limits of volcanic gas monitoring, providing both higher sensitivity and greater chemical resolution in airborne halogen measurements.

## Materials and methods

### Reagents and standards

All experiments were conducted using analytical-grade reagents. Sodium hydroxide monohydrate (99.996%), sodium chloride (99.99%), sodium bromide (> 99%), sodium fluoride (97%), and sodium sulphate (99%) were dissolved in ultrapure water (18.2 MΩ·cm) to prepare calibration standards and absorbing solutions for the alkaline trap. Aqueous SO_2_ solution (5%) was used for evaluating SO_2_ collection, and hydrogen peroxide (> 30%) together with manganese dioxide (99%) served during the post-sampling oxidation procedure. A protonated cation exchange resin (AmberLite Mac-3H) was regenerated with dilute nitric acid (0.01 M) and used for neutralising alkaline solutions prior to ion chromatographic analysis. Standards for GC-HRMS quantification of derivatised halogens included Cl-TMB, Br-TMB, I-TMB, stilbene dichloride (S-Cl_2_), stilbene dibromide (S-Br_2_), and stilbene bromochloride (S-BrCl). All were dissolved in acetone (GC grade) and stored in amber vials at 5 °C.

### Alkaline trap and operation

**Fig. 1:**
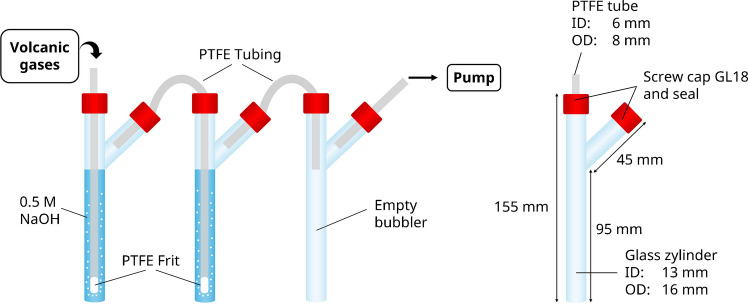
Schematic representation of the alkaline trap (“Bubbler”) with some technical details

The alkaline trap used in this study was designed as a miniaturised alternative to classical impingers with the goal of reducing sample volume and weight during UAV deployment. Each trap consisted of a borosilicate vial equipped with a screw cap through which a 4 µm polyethylene frit (HPLC solvent filter, Techlab) was inserted. The frit was connected to the inlet line. For each experiment, two bubblers were filled with 4 mL of 0.5 M NaOH, while a third bubbler—connected downstream—remained empty to act as a safety trap in case of liquid entrainment. The setup of the alkaline trap (“Bubbler”) is shown with some technical details in Fig. 1. Sampling in the laboratory was typically performed at 250–500 mL min^−1^ for 15–30 min and supplied by Sensidyne GilAir Plus pumps for all laboratory experiments. For UAV-based field deployments, lightweight membrane pumps were employed to minimise payload and power consumption while maintaining sufficient and stable flow rates for gas sampling [36]. During operation, the frit remained fully submerged to ensure fine bubble formation and efficient gas–liquid transfer. After sampling, 1 mL of the alkaline solution was oxidised by addition of 2 µL of hydrogen peroxide for 24 h at room temperature. Excess peroxide was removed by adding a small amount of MnO_2_ and allowing the suspension to react for 48 h. Finally, samples were neutralised by cation exchange resin that had been prewashed until anion-free.

Ion chromatographic measurements were performed using a Dionex ICS-1100 equipped with an AS14A analytical column and precolumn, 4 mm AERS suppressor (55 mA), a 100 µL injection loop, and an autosampler. The eluent consisted of a carbonate/bicarbonate mixture (1 mM NaHCO_3_ + 8 mM Na_2_CO_3_) delivered at 1.0 mL min^−1^. The detector was set at 40 °C, and all samples and standards were analysed at least in duplicate. Chromeleon 7.1.2 was used for peak integration.

### Chlorine permeation source

**Fig. 2:**
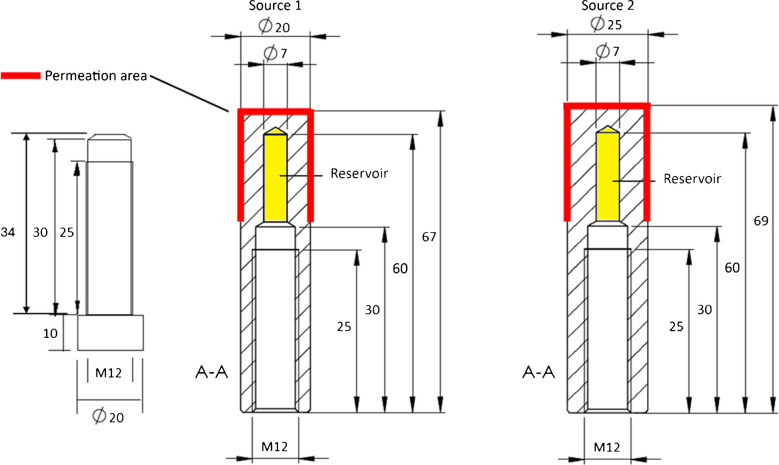
Chlorine permeation tubes, Source 1 (left) and Source 2 (right), all dimensions in mm, PTFE source (hatched area), chlorine reservoir (yellow), active permeation area (red)

A reliable Cl_2_ standard was essential for filter characterisation and a new source had to be developed, since the techniques used for Br_2_ and I_2_ gas generation could not be applied to chlorine [37]. Therefore, two PTFE permeation sources were constructed (Fig. 2) and filled with pre-cooled liquefied chlorine. Chlorine gas was obtained by adding concentrated hydrochloric acid dropwise to potassium permanganate in a glass flask. The evolving gas was continuously flushed by a gentle nitrogen stream into a glass collection tube placed in a −70 °C cooling bath, where the gas condensed as a yellow liquid. After filling, both permeation tubes were sealed with PTFE plugs. Their permeation behaviour was characterised gravimetrically. Each device was placed in a temperature-controlled chamber at 30 °C and continuously flushed with nitrogen at 50 mL min^−1^. The mass was recorded at weekly intervals for several months to determine both the initial equilibration phase and the long-term stable output rate.

#### Safety considerations

The preparation and handling of chlorine permeation sources involving condensed Cl₂ were performed exclusively in a certified chemical fume hood. Chlorine generation, condensation, filling, storage, and characterisation were carried out under continuous ventilation. Personal protective equipment included safety goggles, laboratory coat, chemically resistant gloves, and insulated gloves for cryogenic handling. Leak integrity was verified gravimetrically by weighing each permeation source immediately after filling and again after 24 h. No abnormal mass loss was observed. During the entire study, the sources were stored in a fume hood. For gravimetric measurements, the sources were briefly removed. Potential chlorine release during this step is negligible due to low permeation rates and short handling times.

All procedures complied with institutional laboratory safety regulations. No external transport of liquefied chlorine or special permits beyond standard laboratory safety training were required.

### Filter preparation, measurement, and chamber experiments

Syringe filters (Macherey–Nagel GF-100/25, 25 mm diameter, 1 µm pore size) were used as substrates for the derivatisation coatings. For cis-stilbene filters, 150 µL of a 30 mM solution of cis-stilbene (0.81 mg ± 2%, defined by pipetting precision) in acetone was applied to each filter. The solvent was allowed to evaporate under a gentle stream of laboratory air. The filters were used immediately or sealed and stored at 5 °C for no longer than 3 days to prevent isomerisation. To explicitly assess the shelf life and potential cis-to-trans isomerisation of cis-stilbene on the filter material, a dedicated storage experiment was performed. A cis-stilbene-loaded filter was stored for three days at room temperature, subsequently extracted and analysed by GC, and compared to a freshly prepared filter. Within the analytical precision of the method, no statistically significant loss of the cis-stilbene signal was observed, indicating sufficient short-term stability on the filter under ambient conditions (see data repository). TMB filters were prepared analogously using 30 mM TMB (0.76 mg ± 2%) in acetone; in contrast to cis-stilbene, TMB is not prone to isomerisation and could therefore be stored at room temperature without detectable changes. After sampling, filters were placed in glass vials and extracted four times with 300 µL of acetone (for stilbene filters) or toluene (for TMB filters). Combined extracts were evaporated under nitrogen at 35 °C using a TurboVap system and reconstituted in 200 µL acetone before analysis. The extracts were sonicated twice for 5 min to ensure complete dissolution.

Analyses were performed using a Thermo TRACE 1610 gas chromatograph coupled to an Orbitrap Exploris high-resolution mass spectrometer operating in electron-impact mode (70 eV). A programmed-temperature-vaporisation (PTV) injector was employed minimising thermal decomposition. For the cis-stilbene products, the injector temperature was initially held at 70 °C and then ramped to 200 °C at 14.5 °C min^−1^. The oven was operated according to the following programme: 40 °C for 2 min, 30 °C min^−1^ to 160 °C, then 4 °C min^−1^ to 190 °C (3 min hold), and finally 80 °C min^−1^ to 260 °C (5 min). TMB derivatisation products were analysed using a separate programme starting at 90 °C (3 min), followed by 10 °C min^−1^ to 210 °C, 7 °C min^−1^ to 235 °C, and 20 °C min^−1^ to 250 °C (5 min hold). Quantification was carried out in SIM mode using characteristic fragment ions listed in Table 1. For stilbene derivatives, response factors of anti-isomers were used for quantifying syn-isomers in the absence of authentic standards.

**Table 1:** Characteristic ion fragments and retention times for reaction products of halogens with cis-stilbene and TMB

Filter	Analyte	SIM (selected ion monitoring)	m/z	Retention time (min)
Cis-stilbene	S-Cl_2_	C_7_H_6_Cl	125.0153	14.4 and 14.9
	S-BrCl	C_14_H_12_Cl	215.0622	16.5 and 16.8
	S-Br_2_	C_14_H_12_Br	259.0117	17.5 and 17.7
TMB	Cl-TMB	C_9_H_11_O_3_Cl	202.0391	13.0
	Br-TMB	C_9_H_11_O_3_Br	245.9886	14.0
	I-TMB	C_9_H_11_O_3_I	293.9747	15.0

Collection efficiency experiments were performed in a 5 L dilution chamber through which halogen gases were introduced either in nitrogen (0% RH) or in laboratory air (95 ± 5% RH). Chlorine was introduced using the developed permeation tubes, while bromine and iodine sources followed previous studies [37]; bromine chloride was generated in situ by simultaneous activation of bromine and chlorine sources in the dilution chamber.

**Fig. 3:**
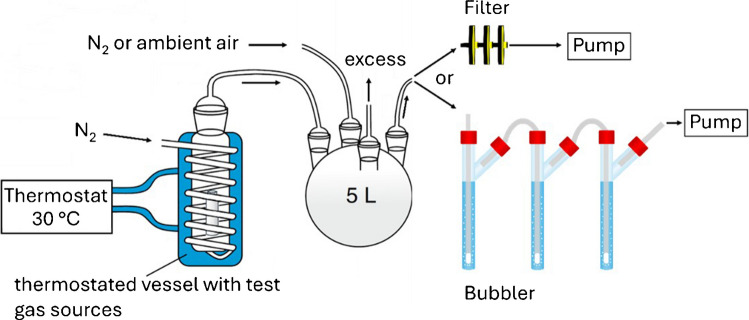
Experimental setup for collection efficiency measurements of alkaline traps and coated filters

Three filters or bubblers were connected in series, and the amount of reaction product on each stage was quantified to evaluate breakthrough behaviour (Fig. 3). Collection efficiencies (CE) were calculated using Eq. 1. Flow rates of 250, 500, and 750 mL min^−1^ were tested. Additional experiments evaluated the influence of humidity, oxygen, and sampling duration. Recovery tests were conducted by applying known amounts of calibration standards directly onto filters, followed by the same extraction and analysis procedure as for sampled filters. The resulting recovery factors were incorporated into the determination of detection and quantification limits.

1 $$\mathrm{CE} \left[\%\right]=\frac{{n}_{1}}{\left({n}_{1}+{n}_{2}+{n}_{3}\right)}*100$$

Any potential coating loss during preparation, laboratory handling, or storage is inherently accounted for by the experimental design. This includes shelf life aspects such as storage at room temperature or at reduced temperature, depending on the coating material. Filters were prepared, stored and handled in the same manner as for field deployment and subsequently subjected to collection efficiency experiments under deliberately extreme laboratory conditions with respect to flow rate, sampling duration and temperature.

### Determination of detection and quantification limits

Limits of detection (LOD) and quantification (LOQ) were determined from calibration statistics. LODs were calculated as 3.3 times the standard deviation of the y-intercept of the calibration curve divided by the slope. LOQs were defined as three times the corresponding LOD. Calibration data used for LOD and LOQ estimation were restricted to the low-concentration range relevant for trace-level detection. This ensures that the derived limits reflect instrumental noise and variability close to the detection threshold rather than signal behavior at higher concentrations. Details on the calibration ranges used for LOD and LOQ determination are provided in “S2”, data repository.

### Drone setup

**Fig. 4:**
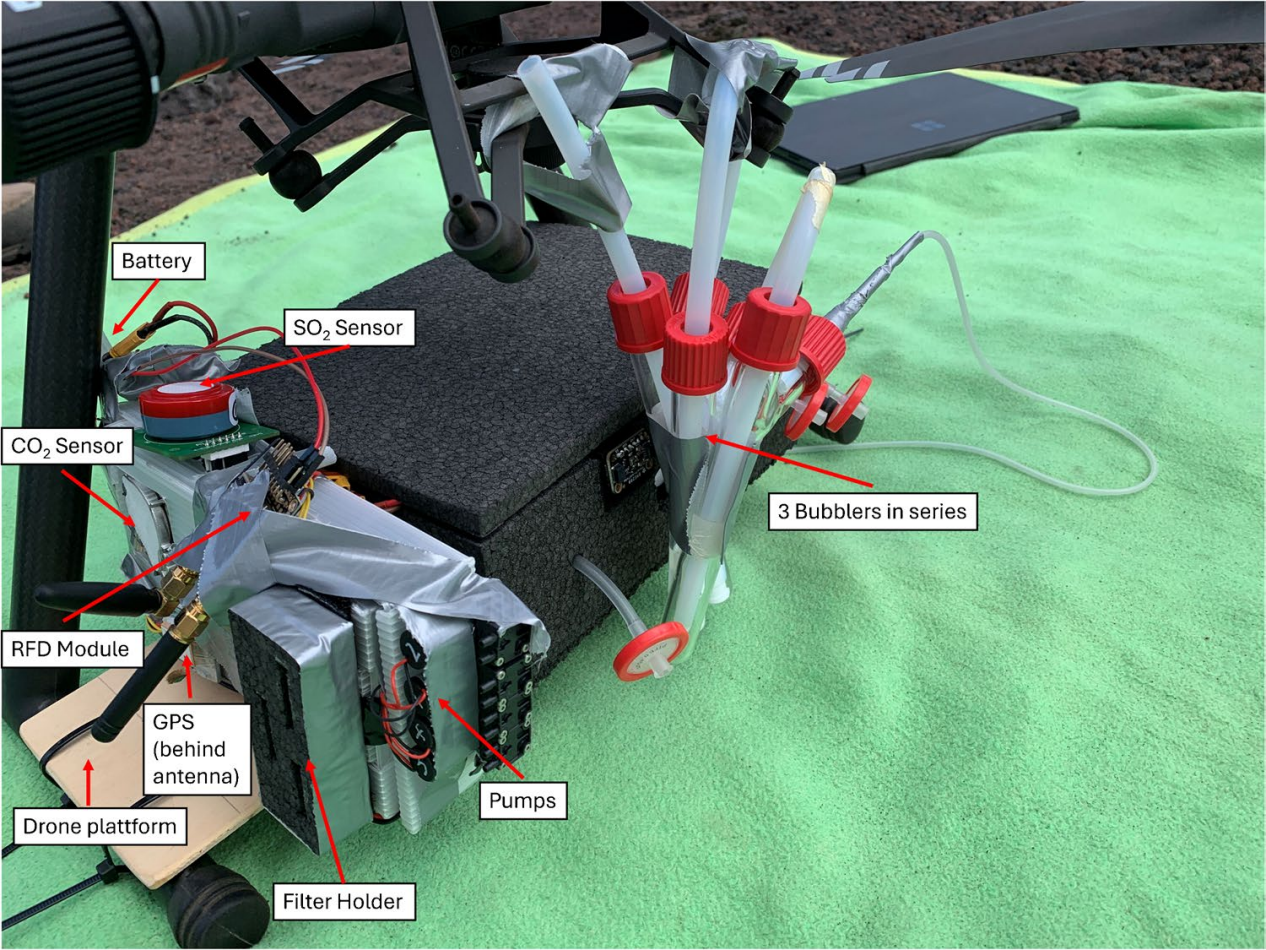
Sampling system with electronics, sensors, communication, pumps, and bubblers (disconnected) mounted on a drone platform. Syringe filters not included

The described sampling system can be integrated into multi-rotor UAV platforms with sufficient payload capacity and flight time, such as the DJI Matrice 350 RTK. A compact sensor module equipped with a gas sensor (e.g. SO_2_) provides real-time information to the operator and can serve as a trigger for activating the sampling sequence [33]. The sampling unit is based on three independent pumps, each of which is responsible for maintaining a constant flow through one of the collection devices (two coated filters, e.g. cis-stilbene and TMB, and one alkaline trap). Pump operation can be remotely controlled, allowing selective activation during the sampling period. The complete system (Fig. 4), sensor unit, control electronics, and pumps was designed, built and modified by Niklas Karbach [36] and with the bubblers and filters has a total weight of approximately 600 g. The sampling system operates autonomously using dedicated batteries and does not draw electrical power from the UAV, any impact on flight time is therefore limited to the additional payload mass. The present study focuses on the development and laboratory validation of UAV-compatible sampling systems. All experiments described here were conducted under laboratory and chamber conditions. No UAV-based field sampling in volcanic plumes was performed within the scope of this work. Consequently, no airspace permissions or field-operation approvals were required for the experiments presented.

## Laboratory results and discussion

### Performance and analytical stability of the alkaline trap

The alkaline trap was developed to achieve quantitative absorption of acidic volcanic gases while minimising weight and sampling volume for UAV-based applications. In controlled dilution chamber experiments (Fig. 3), the system achieved high absorption efficiencies for both weak and strong acidic gases. The collection efficiency tests were performed by evaporating an aqueous SO_2_ solution (5%, 0.43 mL, corresponding to 22 mg SO2) into an ambient air stream containing 550 ± 100 ppm CO_2_ and calculated using Eq. 1. As summarised in Table 2, SO_2_ was captured with an efficiency of 100 ± 1.0%, while CO_2_ exhibited an efficiency of 98 ± 5%. These values confirm that even weakly acidic gases are effectively absorbed, indicating that hydrogen halides (HF, HCl, HBr) will be collected with near-complete efficiency.

**Table 2:** Results of collection efficiency measurements for alkaline traps

Method	Analyte	Flow (mL min^−1^)	Sampling time (min)	Humidity (%)	Collection efficiency (%)
Alkaline trap	SO_2_ (22 mg)	500 ± 25	150	65 ± 10	100 ± 1
	CO_2_ (550 ± 100 ppm, ambient)				98 ± 5

**Fig. 5:**
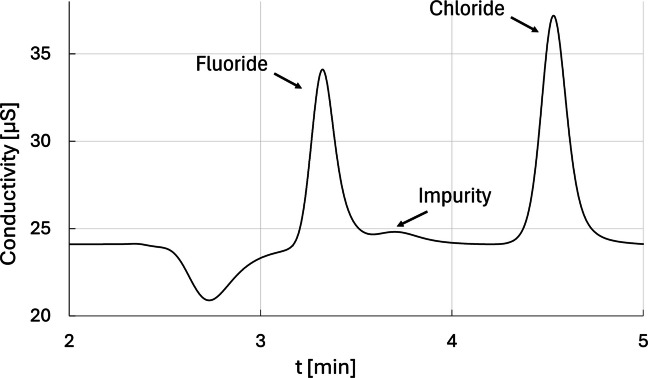
Standard solution of NaF and NaCl in 0.5 M NaOH after sample treatment showing an additional signal at 3.7 min, probably caused by a reaction of hydroxide with the plastic container after two days of storage

A notable observation was the formation of a storage time dependent impurity signal at 3.7 min in the IC chromatogram (Fig. 5), attributed to slow interactions between NaOH and the plastic storage vessel. Glass containers were not considered as an alternative, as concentrated alkaline solutions are known to attack glass surfaces, potentially releasing silicates and introducing additional artefacts relevant for anion analysis.

This impurity did not interfere with fluoride or chloride unless alkaline solutions were stored for extended periods. Storing the alkaline solution at 5 °C significantly slowed the formation of impurities, indicating that the underlying reaction could be reduced but not completely prevented. Consequently, rapid analysis of collected samples is recommended to minimise storage-related contamination. This demonstrates that the alkaline trap provides stable analytical performance under field-relevant storage times.

Recovery experiments confirmed that oxidation (H_2_O_2_) and neutralisation using cation exchange resin do not compromise analytical accuracy. A reference solution and an identical alkaline-treated solution yielded recovery rates of 95 ± 5% for all target analytes. Together with high collection efficiency, this enables accurate quantification of total halogen and total sulphur species, which are critical parameters for deriving ratios such as BrCl/Br_total_ or Br_total_/S_total_ during volcanic monitoring.

The limits of detection and quantification calculated from calibration standards, sample volume, flow rate, recovery and collection efficiency, are listed in Table 3. Resulting LODs ranged from 0.012 µg (HBr) to 1.7 µg (SO_2_), corresponding to sub-ppb to mid-ppb mixing ratios under typical UAV sampling conditions (250 mL min^−1^, 15 min). These values are well suited for near-vent measurements at volcanoes such as Mt. Etna.

**Table 3:** Alkaline trap: LODs and LOQs in absolute amounts and in their mixing ratios at normal conditions using typical sampling settings, flow 250 mL min^−1^, sampling time 15 min

Limit	Alkaline trap “Bubbler”			
	HF	HCl	HBr	SO_2_
LOD (µg)	0.022	0.62	0.011	0.51
LOD (ppbv)	6.6	100	0.84	47
LOQ (µg)	0.066	1.9	0.034	1.5
LOQ (ppbv)	20	310	2.5	140

### Stability and output characteristics of the chlorine permeation source

For characterisation the new chlorine source was stored at 30 °C and weighed once a week. Mass loss curves recorded over several months showed  a clear transition from an initial non-linear permeation phase to a stable, linear regime (see Fig. S2, Supplementary Information). Steady-state output was reached after ~ 6 weeks (Tube 1) and ~ 9 weeks (Tube 2) and remained constant for 4–8 months, depending on the amount of liquid chlorine initially loaded.

Interestingly, both tubes—despite different wall thicknesses—exhibited identical output rates (0.245 and 0.246 mg h^−1^). This is consistent with their nearly identical permeation-area-to-wall-thickness ratios (A/L), the dominant factor controlling permeation [38, 39]. Permeability coefficients derived from the steady-state regime agreed well with literature values (Table 4), confirming that the PTFE devices behave predictably according to established permeation theory [40].

**Table 4:** Permeability of Cl_2_ in PTFE Membranes; Pe = [m^3^(STP) m/(m^2^hbar)] × 10^7^

	A/L (m)	Output rate (mg/h)	P_e_ (this study) at 30 °C, 8.9 bar	P_e_ [40] at 30 °C, 1–3 bar
Source 1	0.37	0.246	0.26	0.13
Source 2	0.36	0.245	0.26	

### Performance of cis-stilbene-coated filters

**Fig. 6:**
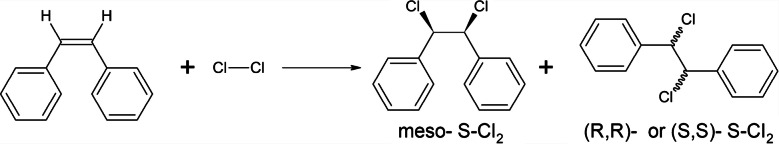
Reaction of cis-stilbene with chlorine, analogous reaction for bromine and bromine chloride

Cis-stilbene reacts with Cl_2_, Br_2_ and BrCl via electrophilic addition (Fig. 6), generating distinct dihalogenated stilbene products. Collection efficiencies determined in the dilution chamber (Table 5) exceeded 99.9% for all tested analytes under extreme conditions (750 mL min^−1^, 30 min). This confirms that cis-stilbene is highly reactive toward molecular halogens and interhalogens.

**Table 5:** Sampling conditions (T = 303.25 K) and collection efficiencies for cis-stilbene and TMB-coated filters

Method	Analyte/concentration	Flow (mL min^−1^)	Sampling time (min)	Humidity (%)	Collection efficiency for all analytes (%)
Cis-Stilbene	Cl_2_ (5.2 ppmv) Br_2_ (60 ppbv)	250 ± 12.5	30	0 (pure N_2_)	100 ± 1
	Cl_2_ (2.6 ppmv) Br_2_ (30 ppbv)	500 ± 25	30	0 (pure N_2_)	100 ± 1
	Cl_2_ (1.7 ppmv) Br_2_ (20 ppbv)	750 ± 37.5	30	95 ± 5 (laboratory air)	100 ± 1
	Cl_2_ (2.6 ppmv) Br_2_ (30 ppbv)	500 ± 25.0	60	0 (pure N_2_)	48 ± 1 (Cl_2_) 65 ± 1 (BrCl) 91 ± 1 (Br_2_)
TMB	Cl_2_ (1.7 ppmv) Br_2_ (20 ppbv) I_2_ (14 ppbv)	750 ± 37.5	60	95 ± 5 (laboratory air)	100 ± 1

At prolonged sampling (60 min) and increased flow (500 mL min^−1^), breakthrough was observed (Cl_2_: 48%, BrCl: 65%), with significantly better retention of Br_2_ (91%). The difference likely reflects both stilbene volatility (leading to partial loss of coating) and the intrinsic reactivity of the analytes. These conditions represent extreme laboratory scenarios; field sampling durations rarely exceed 10–20 min on UAV platforms, ensuring complete collection. Furthermore, the possible reaction of cis-stilbene with hydrogen halides (HCl, HBr, HI), present in significant quantities in volcanic plumes, was tested. Aqueous solutions of these halides (1 mL, 1%) were individually evaporated under a nitrogen stream and passed through cis-stilbene-coated filters. No corresponding reaction products were detected. HF has not been experimentally investigated because working with HF involves considerable experimental and safety restrictions. Given that no addition was observed for HCl, HBr, or HI, and considering the well-known decrease in reactivity from HI to HF in electrophilic additions to alkenes, an addition of HF can be excluded under the investigated conditions.

Experiments with I_2_, ICl, and IBr yielded no detectable products. This observation is consistent with literature reports describing iodine-catalysed cis-to-trans isomerisation of stilbenes, in which thermally labile iodine addition intermediates rapidly decompose to form trans-stilbene [41]. Oxidants such as ozone may affect plume composition upstream of sampling but are not expected to react directly with the coating materials on the timescales relevant for filter sampling. The brominated stilbene products showed pronounced thermal sensitivity and degraded rapidly under conventional split/splitless injection. A PTV injection mode was therefore essential. This ensures that solvent is evaporated before analyte volatilisation, minimising thermal decomposition. Recovery rates of S-Cl_2_, S-BrCl, and S-Br_2_ were 87%, 92%, and 97%, respectively, confirming robust extraction and quantification across all derivatisation products.

### Performance of TMB-coated filters

**Fig. 7:**
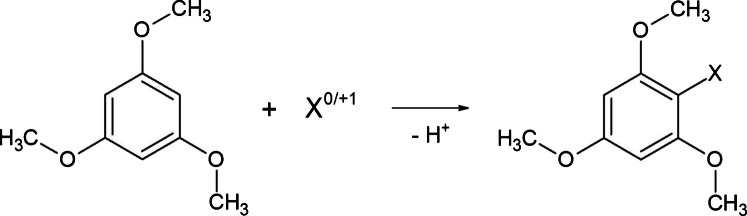
Reaction of TMB with halogen species (X) of oxidation state 0 and +1 yields the corresponding X–TMB adducts

TMB selectively undergoes electrophilic aromatic substitution with halogen species in oxidation states 0 and +1 (Fig. 7). Collection efficiencies were consistently > 99.9% for Cl_2_, Br_2_, BrCl, and I_2_ across various conditions, including low humidity (0% RH in N_2_) and high humidity (95% RH in laboratory air). Table 5 summarises the full efficiency dataset. Recovery rates were 87% (Cl-TMB), 90% (Br-TMB), and 95% (I-TMB).

Possible reactions of TMB with halides were investigated in the same way as with cis-stilbene-coated filters, but no reaction products were detected. In connection with halogen studies in volcanic plumes, the question arises as to what extent reactions with halogen radicals (Br·, BrO·) may also be responsible for the observed product formation. Therefore, TMB filters were exposed to elemental bromine under daylight and dark conditions. Under dark conditions (i.e. without bromine photolysis forming bromine radicals), higher Br-TMB signals were obtained than under photolysis conditions, which rules out major contributions from radical reactions. In addition, it is generally expected that radical substitution on TMB would occur at one of the methyl groups, resulting in the formation of an R-CH_2_Br derivative. However, no such products were observed. The same applies to the reaction of BrO with TMB, which cannot be easily reproduced in the laboratory due to the difficulty of generating defined BrO concentrations. Moreover, a hypothetical electrophilic substitution of BrO on the aromatic ring would require the formation of a hydroxyl radical as the leaving group, making such a reaction mechanistically unfavourable. We therefore rule out any significant contribution from radical bromine compounds (Br· or BrO·) to the measurement results obtained using the method presented.

### Detection limits and mixing ratios for all filter systems

Detection limits for both filter types are summarised in Table 6. For stilbene filters, LODs ranged from 0.12 ng (BrCl) to 0.30 ng (Cl_2_); TMB filters achieved similar sensitivities with LODs between 0.060 ng (I_2_) and 0.16 ng (Br_2_).

**Table 6:** Coated filters: determined LODs and LOQs in absolute amounts and in their mixing ratios at normal conditions using typical sampling conditions, flow 250 mL min^−1^, sampling time 15 min at 1 atm

Limit	Stilbene filter			TMB filter		
	Cl_2_	BrCl	Br_2_	Cl_2_	Br_2_	I_2_
LOD (ng)	0.30	0.12	0.19	0.094	0.16	0.060
LOD (pptv)	7.1	2.7	3.3	2.2	3.7	1.1
LOQ (ng)	0.90	0.35	0.56	0.28	0.48	0.18
LOQ (pptv)	21	8.0	9.8	6.7	11	3.2
For typical UAV sampling (250 mL min^-1^, 15 min), these values correspond to: Cl_2_: 7.1–21 pptv BrCl: 2.7–8.0 pptv Br_2_: 3.3–9.8 pptv I_2_: 1.1–3.2 pptv						

These detection limits represent a 1–2 orders of magnitude improvement over previously published denuder systems [33] and enable detection of reactive halogen species at concentrations typical of ageing volcanic plumes. Field measurements of reactive bromine species using TMB-based sampling at Mt Etna have reported mixing ratios in the low-ppbv range [33]. The sub-pptv detection limits achieved here are therefore well below concentrations observed close to the vent and demonstrate sufficient sensitivity for near-source plume conditions.

For other molecular halogen species targeted by the cis-stilbene filters, quantitative concentration data in volcanic plumes are generally scarce, one study of Cl_2_ reporting ppmv concentration [23]. The achieved detection limits indicate that these species can be addressed across a wide range of plume conditions, although interpretation of absolute mixing ratios remains challenging due to strong spatial and temporal variability and plume dilution during UAV-based sampling.

## Conclusions

The presented methods were evaluated for their performance and optimised for rapid analyte enrichment, minimal weight, short sampling times, and high analytical sensitivity. The alkaline trap represents a miniaturised alternative (optimised weight, sample volume and sample treatment) over existing techniques, in which the strengths of the established approaches are combined, specifically designed for UAV-based deployment, still enabling the quantification of total halogen and sulphur species for realistic plume conditions (e.g. Mt Etna volcanic plume). Absolute detection and quantification limits were 0.022 and 0.066 µg for HF, 0.62 and 1.9 µg for HCl, 0.011 and 0.034 µg for HBr, and 0.51 and 1.5 µg for SO_2_.

Cis-stilbene-coated syringe filters allow selective quantification of chlorine, bromine, and bromine chloride, with absolute detection and quantification limits of 0.30 and 0.90 ng for Cl_2_, 0.12 and 0.35 ng for BrCl, and 0.19 and 0.56 ng for Br_2_. TMB-coated filters, adapted from recently developed denuder systems and combined with GC-HRMS analysis, provide improved detection limits for reactive halogen species in oxidation states 0 and + 1. Absolute detection and quantification limits for TMB filters were 0.094 and 0.28 ng for chlorine, 0.16 and 0.48 ng for bromine, and 0.060 and 0.18 ng for iodine.

By combining total halogen quantification using the alkaline trap with selective speciation of reactive halogen species using derivatisation filters, the presented UAV-compatible approach enables direct and simultaneous assessment of total and oxidised halogen fractions in volcanic plumes. This provides the methodological basis for determining widely used volcanological ratios such as Br(total)/S(total), Br(reactive)/Br(total), BrCl/Br(total), or (Br_2_ + BrCl)/Br(total) under near-vent conditions, which are key parameters for interpreting halogen activation, plume chemistry, and volcanic degassing processes.

Plume sampling can be hazardous, and certain locations, in particular aged plumes, (minutes after the gas emissions) are usually inaccessible. UAV-based sampling offers a viable solution although it is limited by payload capacity and sampling time. Consequently, short sampling durations and weight-optimised devices are highly advantageous. The study of gas- and particle-phase processes in volcanic plumes, where rapid chemical transformations occur, would be virtually impossible without UAVs. In the future, autonomous drone missions with onboard sensors could enable remote volcano monitoring and direct measurements.

Several limitations of the methods presented should be considered. Brominated stilbene reaction products exhibit pronounced thermal sensitivity, which necessitates mild injection conditions such as programmed-temperature vaporisation and represents an analytical constraint. The coated filter systems are selective and differ in their chemical scope. Cis-stilbene selectively traps molecular halogens and interhalogens while other halogen species are not retained. TMB reacts with halogen species in oxidation states 0 and +1; however, the exact reaction pathways and potential contributions from other reactive halogen species could not be independently verified. Radical halogen species are not actively trapped by either coating and may therefore contribute to the overall halogen budget without being reflected in the speciation results. Furthermore, UAV-based sampling is inherently limited in sampling duration and spatial coverage. Volcanic plumes are often highly heterogeneous in space and time, and the measured concentrations therefore represent local plume snapshots rather than fully integrated plume compositions. The present study provides a laboratory-based benchmark for UAV-compatible halogen sampling. Application under real volcanic plume conditions represents the next step to assess performance under atmospheric complexity beyond controlled environments.

Future research should explore improved analytical techniques for detecting inorganic halogens in basic solutions that can achieve detection limits comparable to the presented filter methods. This is particularly relevant because bromine concentrations in volcanic plumes are often close to or below the detection limits of conventional ion chromatography, and iodine, which occurs at even lower levels, is practically undetectable by IC in alkaline solution. Successful determinations of bromine and iodine by ICP–MS have already been demonstrated, providing a sensitive approach for total halogen quantification. Alternatively, conversion of inorganic halogens into organic derivatives suitable for GC–MS analysis could offer an efficient complementary method.

## Supplementary Information

Below is the link to the electronic supplementary material.

Supplementary file1 (DOCX 533 KB)

## Data Availability

The raw data supporting this study, including raw data with mass spectra for each stilbene and TMB reaction product, low level calibration files, raw data that support storage behaviour of cis-stilbene filters and permeation mass loss time series are available under: 10.5281/zenodo.18417414. Additional mass spectra for each product were added in the Supplemental Information.
