# Supplementary material for: On-filter derivatisation and alkaline trap sampling for UAV-based gas-phase halogen speciation in volcanic plumes
Source: Anal Bioanal Chem. 2026 Feb 26;418(16):5193–204. doi: 10.1007/s00216-026-06403-7 (PMC13423908; doi:10.1007/s00216-026-06403-7)
Supplement: Supplementary file 1 — Supplementary file1 (DOCX 533 KB) [file 216_2026_6403_MOESM1_ESM.docx]

Supplemental Information

Contents

[1 Standard preparation 2](#_Toc220594134)

[1-Iodo-2,4,6-trimethoxybenzene (I-TMB) 2](#_Toc220594135)

[1-bromo-2-chloro-1,2-diphenylethane (Stilbene bromide chloride, S-BrCl) 2](#_Toc220594136)

[2 Extraction process filter 2](#_Toc220594137)

[3 Output rate Chlorine Source 3](#_Toc220594138)

[4 GC-Measurements 3](#_Toc220594139)

[4.1 HRMS-Spectra 4](#_Toc220594140)

[5 Calibration, LOD/LOQ and Weighing 11](#_Toc220594141)

[6 Recovery rates 11](#_Toc220594142)

[Alkaline trap 11](#_Toc220594143)

[Cis-stilbene 11](#_Toc220594144)

[TMB 11](#_Toc220594145)

## 1 Standard preparation

### 1-Iodo-2,4,6-trimethoxybenzene (I-TMB)

2 mmol of each 1,3,5-Trimethoxybenzene (0,336 mg) and Iodine (0.508 g) were solved in 20 mL glacial acetic acid and stirred at RT for 48 hours in the dark. 1 Liter of pure water was added until there was no more precipitation. The residue was filtered, solved in 15 mL of DCM and washed three times with 10 mL of 0.01 M NaOH followed by three times with 10 mL water. The solution was dried with 2 g of MgSO_4_. The organic solvent was evaporated under reduced pressure. The solid was recrystallized two times using 5 mL of ethanol. The yellow crystals were characterized by GC-HRMS m/z: 294.9747 (M+); 0.48 g (81 %).

### 1-bromo-2-chloro-1,2-diphenylethane (Stilbene bromide chloride, S-BrCl)

19 mmol of trans-stilbene (3.5 g) and 19 mmol of N-bromoacetamide (2.6 g) were solved in 100 mL MTBE and 50 mL Ethanol and stirred for 30 min. 4 mL HCl (30 %) was added dropwise. The solution first turned yellowish and became clear after stirring for 1 hour. A colorless solid precipitated after colling to – 20 °C. The solid was filtered, washed with cool water (3 x 10 mL), recrystallized in petroleum ether and characterized by GC-HRMS m/z: 215.0622 (C14H10Br^+^), 168.9648 (C7H6Br^+^); 3.82 g (68 %)

## 2 Extraction process filter


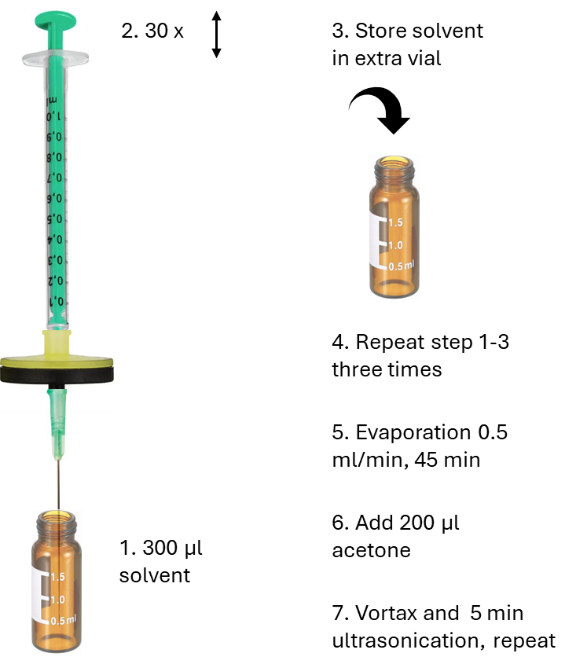


Figure S1: Extraction process scheme for derivatization filters.

Fill 300 µL of solvent in a GC-Vial, extract the solvent thirty times through the filter with a syringe and transfer the solution into another GC-Vial. Repeat this step again 3 times to a total of 1.2 ml of solution. Evaporate the solvent, add 200 µL of acetone. Vortex the solution and sonicate it for 5 min, repeat this step.

## 3 Output rate Chlorine Source


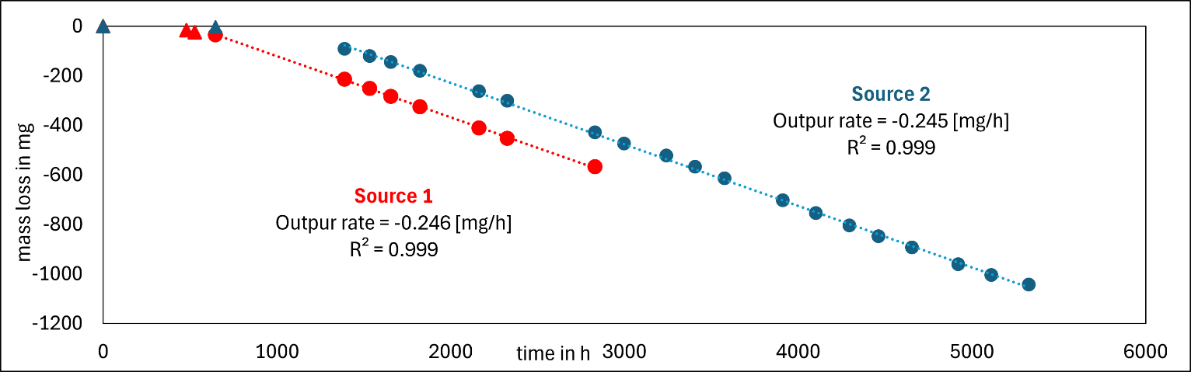


Figure S2: Mass loss of chlorine permeation sources with corresponding output rates. Triangles: non steady state.

## 4 GC-Measurements

**PTV**

| PTV (Stilbene) | Rate [°C/s] | T [°C] | Hold time [min] | Split flow [mL/min] |
| --- | --- | --- | --- | --- |
| Start  Injection  Transfer  Cleaning | 14.5  14.5 | 70  70  200  280 | 3  1  1 | 50 |

**Oven program**

| Time [min] | Rate [°C/min] | T [°C] | Hold time [min] |
| --- | --- | --- | --- |
| 2  6  16.5  22.4 | 0  30  4  80 | 40  160  190  260 | 2  0  3  5 |

**PTV**

| PTV (TMB) | Rate [°C/s] | T [°C] | Hold time [min] | Split flow [mL/min] |
| --- | --- | --- | --- | --- |
| Start  Injection  Transfer  Cleaning | 14.5  14.5 | 270  270  270  310 | 0  1  3 | 100 |

**Oven program**

| Time [min] | Rate [°C/min] | T [°C] | Hold time [min] |
| --- | --- | --- | --- |
| 0  15  18.6  24.3 | 0  10  7  20 | 90  210  235  250 | 3  0  0  5 |

### 4.1 HRMS-Spectra

**Stilbene derivates Chromatogramm, chamber experiments**

**
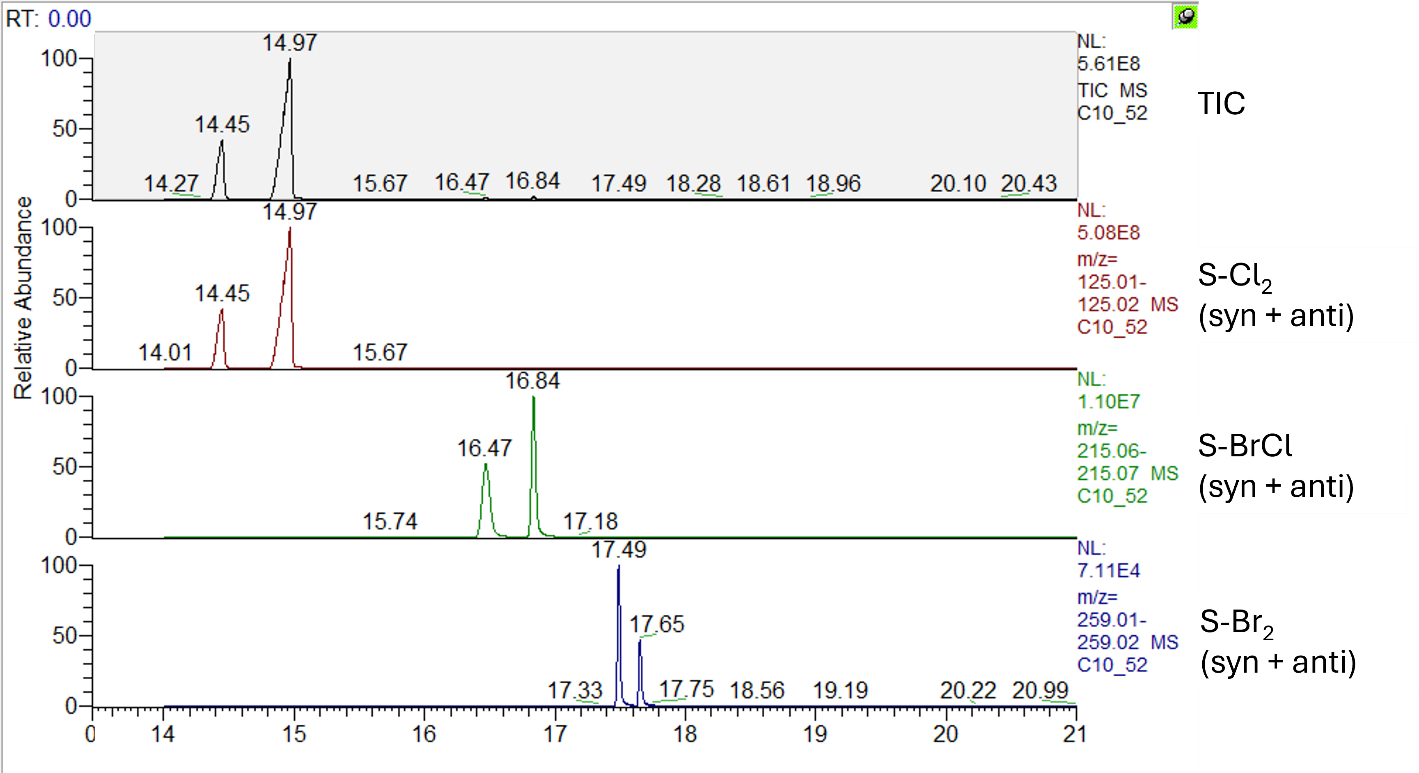
**

**S-Cl_2_**


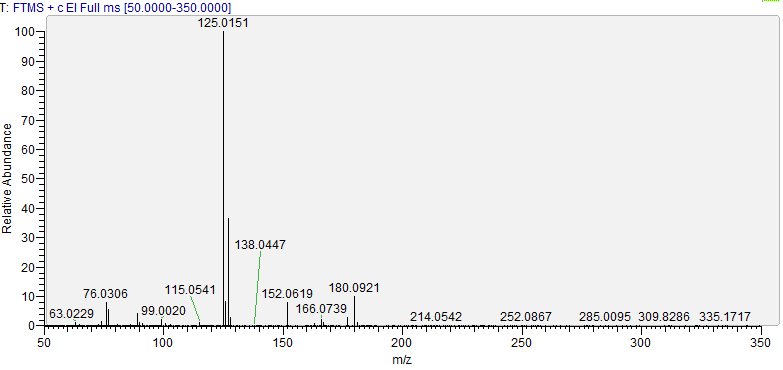


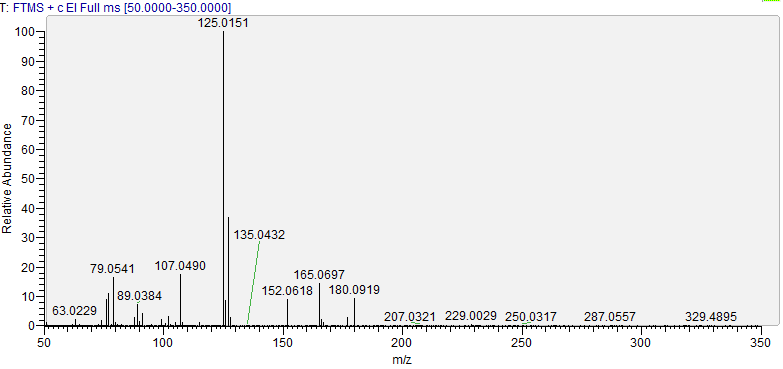


Figure 3: HRMS of S-Cl2 (Retention time: 14.4 (top) and 14.7 min (bottom) with characteristic mass fragments.

m/z: 125.0151 C7H6^35^Cl+

127.0122 C7H6^37^Cl+

180.0919 C14H12+

165.0697 C13H9+

152.0618 C12H8+

**S-BrCl**


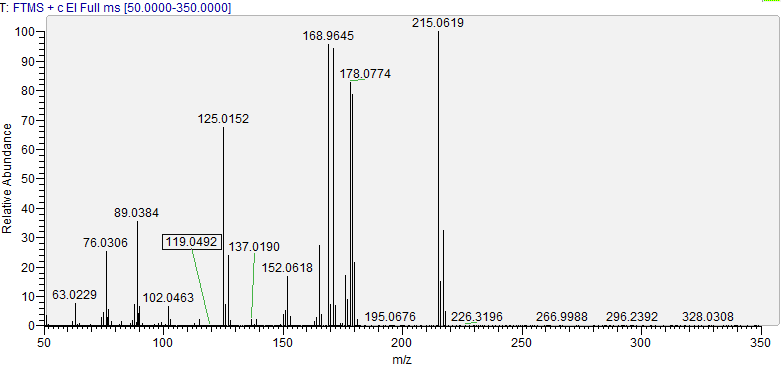


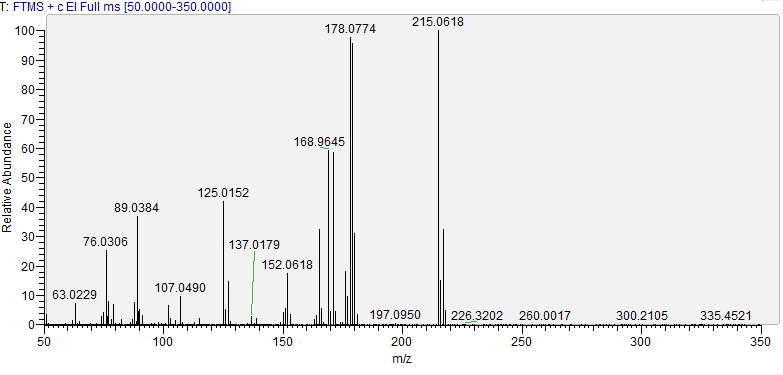


Figure 4: HRMS of S-BrCl (Retention time: 16.5 (top) and 16.8 min (bottom) with characteristic mass fragments.

m/z: 215.0618 C14H12^35^Cl+

217.0590 C14H12^37^Cl+

168.9645 C7H6^79^Br+

170.9624 C7H6^81^Br+

125.0151 C7H6^35^Cl+

127.0122 C7H6^37^Cl+

178.0774 C14H10+

152.0618 C12H8+

**S-Br_2_**


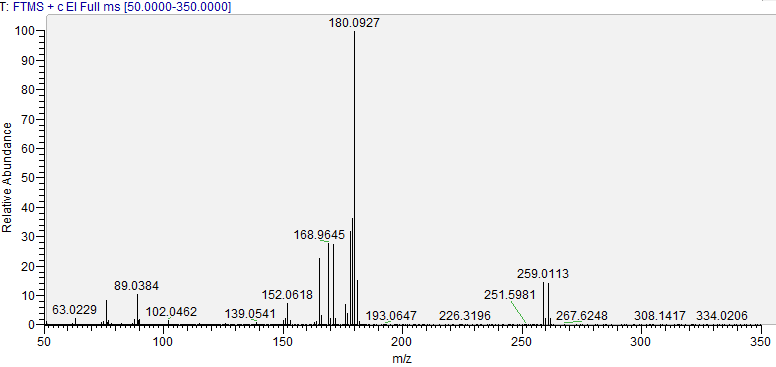


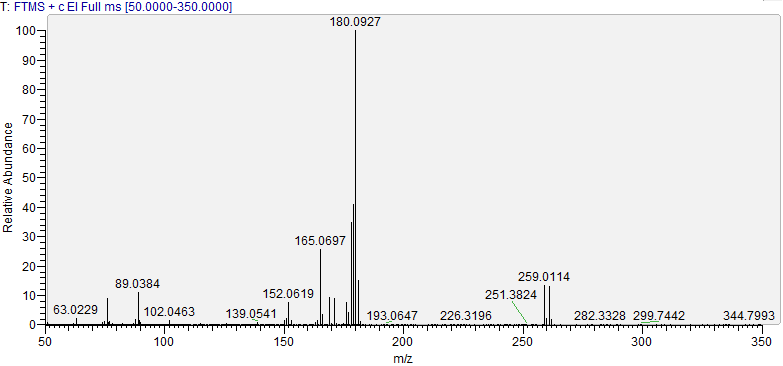


Figure 5: HRMS of S-Br2 (Retention time: 17.5 (top) and 17.7 min (bottom) with characteristic mass fragments.

m/z: 215.0618 C14H12^79^Br+

217.0590 C14H12^81^Br+

165.0697 C13H9+

180.0927 C14H12+

152.0618 C12H8+

**TMB derivates Chromatogramm, chamber experiments**

**
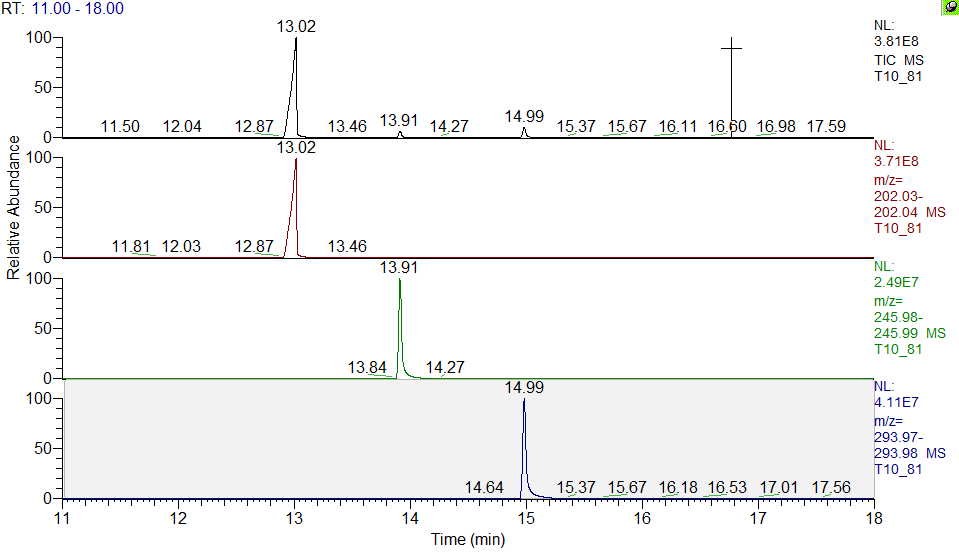
**

**Cl-TMB**


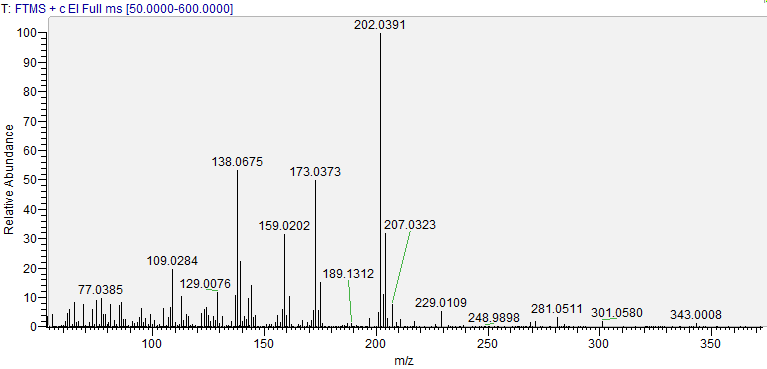


Figure 6: HRMS of Cl-TMB (Retention time: 13.0 min) with characteristic mass fragments.

m/z: 202.0391 C9H11^35^ClO3+

204.0361 C9H11^37^ClO3+

173.0373 C8H10^35^ClO2+

138.0675 C8H10O2+

159.0202 C7H8^35^ClO2+

**Br-TMB**


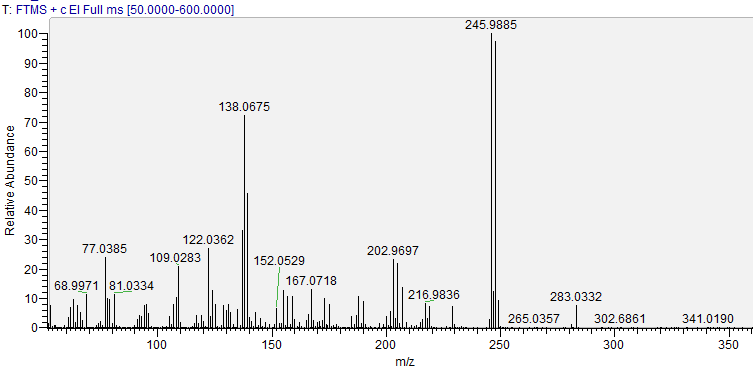


Figure 7: HRMS of Br-TMB (Retention time: 14.0 min) with characteristic mass fragments.

m/z: 245.9885 C9H11^79^BrO3+

247.9864 C9H11^81^BrO3+

202.9697 C7H8^79^BrO2+

204.9684 C7H8^81^BrO2+

138.0675 C8H10O2+

**I-TMB**

**
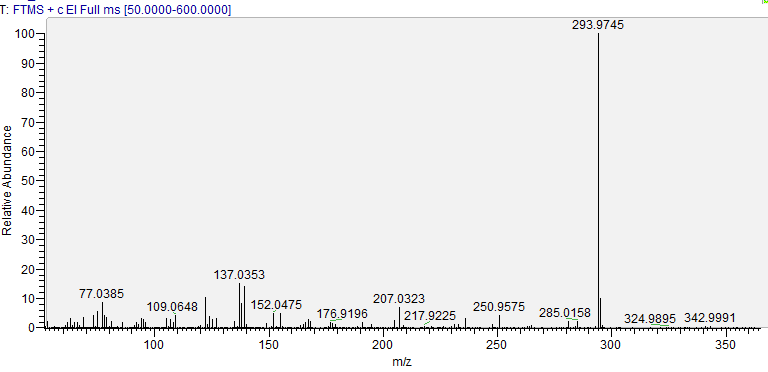
**

Figure 8:HRMS of I-TMB (Retention time: 15.0 min) with characteristic mass fragments.

*m/z: 293.9745 C9H11IO3+*

## 5 Calibration, LOD/LOQ and Weighing

LOD and LOQ values were calculated from calibration statistics using the low-concentration range of the calibration data. For each analyte, calibration points close to the detection threshold were selected to reflect instrumental noise and signal variability under trace-level conditions. Calibration ranges, number of data points and resulting regression parameters used for LOD and LOQ determination are summarised in Excel Sheet S2-Supplemental Information.

## 6 Recovery rates

### Alkaline trap


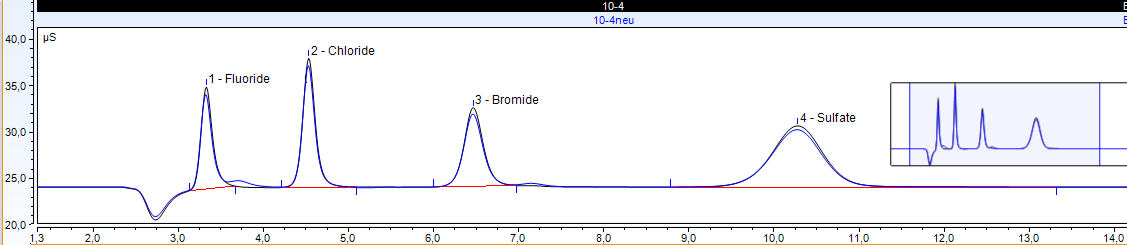


Figure S9: Chromatogram of two analyte solutions. Recovery rates for all analytes is 95 due to dilution with wet cation exchange resin. Comparsion of untreated solution (black) and neutralized solution with the same concentration of analytes (blue)

### Cis-stilbene

| Method | Reaction product | Volumn [µL] | Concentration [µg/L] | Recovery rate [%] |
| --- | --- | --- | --- | --- |
| Stilbene Filter | S-Cl_2_  S-BrCl  S-Br_2_ | 200 | 50 | 85 ± 1  90 ± 1  96 ± 1 |
|  |  |  | 750 | 89 ± 1  94 ± 1  98 ± 1 |

### TMB

| Method | Reaction product | Volumn [µL] | Concentration [µg/L] | Recovery rate [%] |
| --- | --- | --- | --- | --- |
| TMB Filter | Cl-TMB  Br-TMB  I-TMB | 200 | 50 | 82 ± 1  88 ± 1  97 ± 1 |
|  |  |  | 750 | 92 ± 1  91 ± 1  92 ± 1 |
